# Glutathione oxidation in cerebrospinal fluid as a biomarker of oxidative stress in amyotrophic lateral sclerosis

**DOI:** 10.1186/s40035-025-00496-3

**Published:** 2025-07-07

**Authors:** Trong Khoa Pham, Nick Verber, Martin R. Turner, Andrea Malaspina, Mark Oliver Collins, Richard J. Mead, Pamela J. Shaw

**Affiliations:** 1 Sheffield Institute for Translational Neuroscience (SITraN), University of Sheffield, 385 Glossop Road, Sheffield, S10 2HQ UK; 2 School of Biosciences, University of Sheffield, Sheffield, S10 2TN UK; 3 biOMICS Facility, Faculty of Science Mass Spectrometry Centre, University of Sheffield, Sheffield, S10 2TN UK; 4 Neuroscience Institute, University of Sheffield, Sheffield, UK; 5 NIHR Sheffield Biomedical Research Centre, Sheffield, UK; 6 Nuffield Department of Clinical Neurosciences, John Radcliffe Hospital, Level 6 West Wing, Oxford, OX3 9DU UK; 7 Neuromuscular Department, Motor Neuron Disease Centre, Queen Square Institute of Neurology, University College London, London, UK

Neurodegenerative diseases, such as amyotrophic lateral sclerosis (ALS), are devastating conditions, and existing approaches have made few inroads towards the goal of slowing the progression of this disease. Oxidative stress is considered a crucial factor directly or indirectly involved in several neurodegenerative diseases [1, 2]. We set out to develop biochemical biomarkers of target engagement (a direct measure of drug action) to measure the effects of therapies targeting oxidative stress, including edaravone and nuclear factor erythroid 2-related factor 2 (NRF2) activators [3], which show promise as disease-modifying agents for neurodegenerative diseases. Such ‘translational biomarkers’ provide a link between preclinical models and human clinical trials, facilitate dose selection, speed up clinical therapeutic development, provide early clinical proof-of-concept and are now seen as critical parameters for successful drug development [4]. In addition, molecular biomarkers of disease progression are needed to assess the efficacy of new treatments for ALS. Since the brain is a difficult organ to access, changes in the abundance or oxidation state of molecules can be measured in biofluids such as cerebrospinal fluid (CSF).

To maximise the use of valuable CSF samples, we developed a mass spectrometry (MS)-based workflow for measuring protein abundance, protein oxidation, and glutathione oxidation in the same sample. In a typical proteomic analysis of CSF, immunodepletion is performed to remove abundant proteins and reduce the high dynamic range of this biofluid. A concentration step is often included before immunodepletion, generating two fractions: a concentrated protein fraction for immunodepletion and a low-molecular-weight fraction typically discarded (Fig. 1a). We have developed a targeted MS method with the required sensitivity to measure glutathione in this lower-molecular-weight fraction, allowing measurement of protein abundance and oxidation as well as the redox state of glutathione, all from the same aliquot of CSF.

**Fig. 1:**
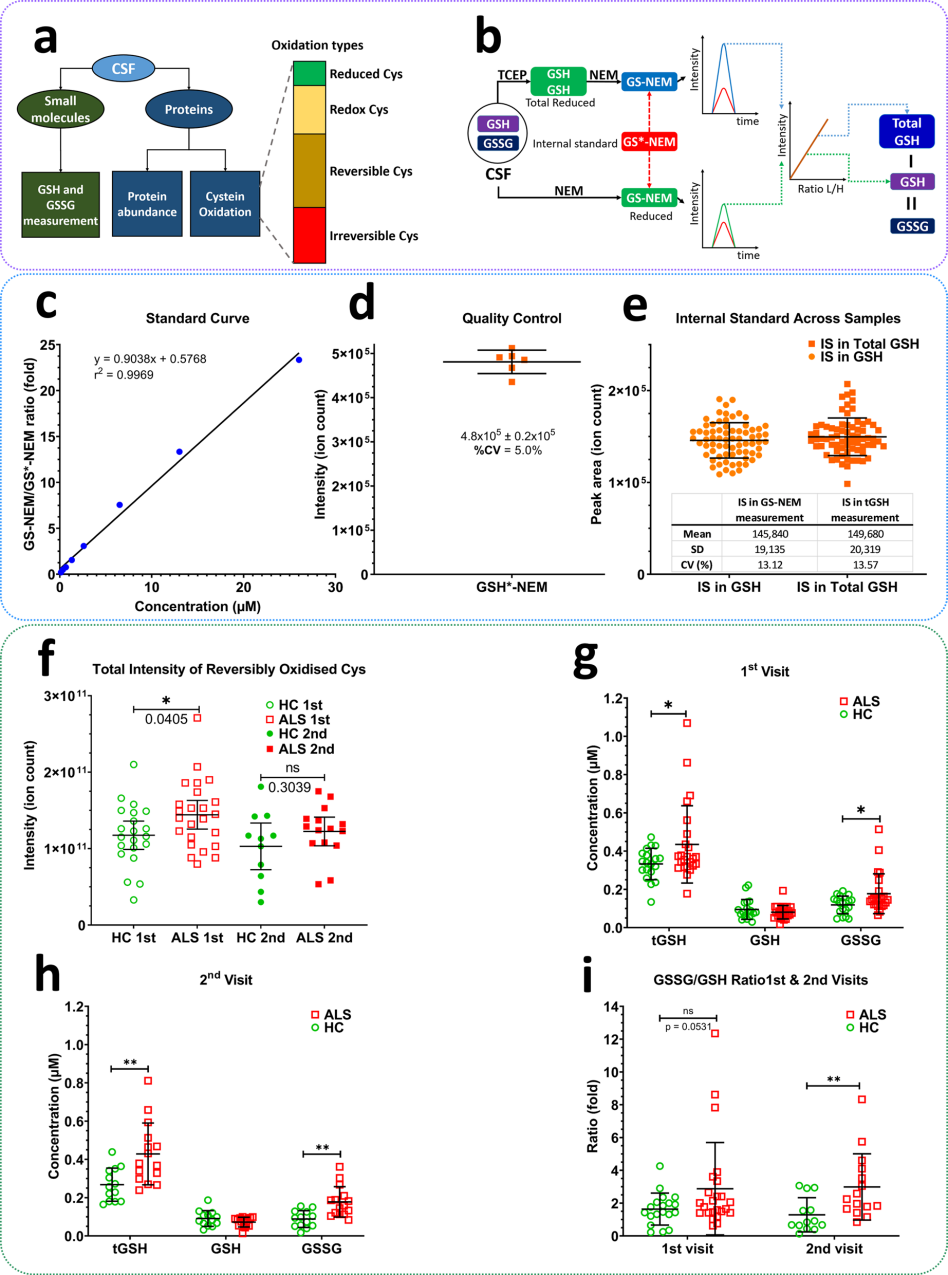
**a** Oxidation types measured using our workflow. Reduced glutathione (GSH) and total reduced glutathione (tGSH) are measured using a targeted MS assay in a lower molecular fraction obtained from a protein concentration step. Oxidised glutathione (GSSG) levels are inferred from GSH and tGSH levels. Protein abundance and different types of protein oxidation on cysteine residues are measured by LC–MS/MS analysis. Reduced Cys: Cys residues identified in reduced form only. Redox Cys: Cys residues in both reduced and reversibly oxidised forms; the ratio of oxidised to reduced forms were used for comparisons between ALS patients and healthy controls. Reversible Cys: Cys residues identified in reversibly oxidised form only. Irreversible Cys: Cys residues identified in the irreversibly oxidised form only. **b** A workflow used to analyse glutathione oxidation in CSF samples. While GSH was directly alkylated with NEM, GSSG was first reduced using TCEP before being alkylated with NEM. A stable isotope-labelled internal standard GS*-NEM was added to each sample before MS analysis. GSH and total GSH are measured in separate LC-MRM assays, and levels of oxidised glutathione (GSSG) are inferred from the difference between GSH and total GSH levels. **c** Performance of a targeted MS assay for glutathione oxidation. The standard curve was used to determine concentrations of GS-NEM and tGS-NEM in CSF. **d**, **e** Intensity of heavy isotope-labelled standard (IS) alone (**d**) and in CSF samples (**e**). IS was used for quality control of measurements and normalisation of data. **f** Total intensity of reversibly oxidised Cys (proteins) in HC and ALS groups at first and second visits. The total intensity is the sum of all peptide intensities bearing reversibly oxidised Cys per sample. **g**–**i** Concentrations of GSH, tGSH and GSSG as well as GSSG/GSH ratios in ALS and HC CSF at the first and second visits. ns: not significant, *P < 0.05, **P < 0.01, Welch’s *t*-test

We employed a redox proteomics method (Fig. 1a) to measure different types of cysteine oxidation in CSF samples from ALS patients at two time points 4 months apart (visit 1, *n* = 24 and visit 2, *n* = 15) compared to healthy controls (HCs) (visit 1, *n* = 20 and visit 2, *n* = 11) (Table S1). This approach involves additional sample preparation steps and data analysis compared to a standard analysis for measuring protein abundance. In this way, protein oxidation and protein abundance are measured simultaneously, allowing the normalisation of oxidation levels to protein levels. To maximise coverage of the CSF proteome, we performed immunodepletion of abundant proteins and collected the flow-through for glutathione analysis. Proteins were then enzymatically digested, and the generated peptides were analysed by LC–MS/MS analysis. 1561 proteins (ALS and HC groups) were identified at a 1% false discovery rate, and 699 proteins were quantified in 70% of samples (Additional file 1: Table S2, Additional file 2: Table S3). Multiple ANOVA testing revealed five proteins significantly increased in abundance in ALS compared to HCs in both visits, including chitotriosidase-1 (CHIT1), CHI3L1 (chitinase-3-like protein 1), CHI3L2 (chitinase-3-like protein 2), COL18A1 (collagen alpha-1(XVIII) chain) and FCGR3A (low affinity immunoglobulin gamma Fc region receptor III-A). No significant changes were observed in the abundance of these proteins in ALS CSF between the two consecutive visits. Furthermore, 9 and 14 proteins were significantly more abundant in ALS at the first and the second visits, respectively (Table S2).

The abundance of reduced, reversibly and irreversiblely oxidised cysteine-containing peptides from ALS vs HC groups was compared to estimate the ratio of cysteine oxidation. There were 47, 505, 531 and 63 cysteine sites detected as reduced only, redox (reduced and oxidised forms), reversible (oxidised only) and irreversible, respectively. DPP7 (dipeptidyl peptidase 2), CFB (C3/C5 convertase) and BCAN (brevican core protein) exhibited increased oxidation in ALS compared to HCs at both visits (Additional file 3: Table S4). In the ALS group, the oxidation of some proteins was increased between visits but was not significant compared to the HC groups (at either visit). However, there was a significant increase in the total intensity of reversibly oxidised Cys (the sum of all oxidised peptide intensities) between the ALS and HC groups at the first visit (1.23 fold, *P* = 0.0405), but this increase was not statistically significant at the second visit (1.19 fold, *P* = 0.3039) (Fig. 1f).

Several MS methods have been developed to measure glutathione levels in blood and other biological samples (Additional file 4: Table S6). However, to our knowledge, there are no previous reports using this approach to measure glutathione in CSF. The level of glutathione in CSF is significantly lower than in other biofluids and, therefore, its detection requires the higher sensitivity afforded by nano-flow chromatography. We developed a highly sensitive, targeted nano-flow LC–MS/MS-based multiple reaction monitoring (MRM) method to measure glutathione levels and the redox state. This involves measuring reduced glutathione (GSH) and total glutathione (tGSH, comprising both free reduced GSH and newly formed reduced GSH generated from oxidised glutathione (GSSG) through reduction with tris(2-carboxyethyl)phosphine [TCEP]) in human CSF (Fig. 1b-e, Table S6). The concentration of GSSG was determined by subtracting GSH from tGSH. The method employs an alkylation step using N-ethylmaleimide (NEM) to derivatise GSH into GS-NEM, which is more stable for storage and enhances the sensitivity of detection of this derivatised compound by MS. A heavy stable isotope-labelled GS*-NEM was used as an internal standard in the processed samples to achieve accurate quantitation and data normalisation (Figs. S1 and S2).

Concentrations of GSH, tGSH and GSSG in CSF were determined by GS-NEM and tGS-NEM measurements, and the detailed results are shown in Fig. 1g, h and Tables S5 and S6. There were no significant differences in GSH between the ALS and HC groups at either the first or the second visit, or between visits. However, tGSH in the ALS group was significantly higher than that of the HC group for the first (1.33-fold, *P* = 0.0215) and the second (1.50-fold, *P* = 0.0143) visits. Similarly, GSSG was significantly increased in ALS compared to HC groups for the first (1.54-fold, *P* = 0.0041) and the second (2.0-fold, *P* = 0.0018) visits. The GSSG/GSH ratio in the ALS group was also significantly higher than that in the HC group at the second visit (2.84 vs 1.33, *P* = 0.0120). The mean concentrations of both tGSH and GSSG in the CSF of ALS patients were almost unchanged between the first and the second visits (0.44 ± 0.2 μmol/L vs 0.43 ± 0.16 μmol/L for tGSH, and 0.18 ± 0.1 μmol/L vs 0.18 ± 0.08 μmol/L for GSSG).

We employed Pearson correlation and simple linear regression models to investigate the correlations between potential biomarkers and clinical parameters of ALS patients (Additional file 5: Table S7). Four proteins at the first visit (CFD [complement factor D], *P* = 0.0436; CFH [complement factor H], *P* = 0.0330; FCGR3A, *P* = 0.0021; SERPINA3 [alpha-1-antichymotrypsin], *P* = 0.0051) and two proteins at the second visit (PKM, *P* = 0.0344; CHIT1, *P* = 0.0134) were positively correlated with the disease progression rate of ALS patients. Among these proteins, SERPINA3 also negatively correlated with the ALSFRS-R score (*P* = 0.0444) at the first visit. Finally, VGF positively correlated with symptom onset (*P* = 0.0284). There was also a positive correlation between the abundance of CA11 and the progression rate in ALS patients (*n* = 7) between two visits (*P* = 0.0069). Both tGSH (*P* = 0.0055) and GSSG (*P* = 0.0071) concentrations and the GSSG/GSH ratio (*P* = 0.0227) correlated positively with disease duration until the first visit. There were also positive correlations between the total intensity of reversibly oxidised Cys and the ratio of GSSG/GSH in ALS patients for both visits (*P* = 0.0193 and 0.0367, respectively). Furthermore, this correlation was significantly increased between the two visits since a positive correlation was found between these time points (*P* = 0.0405) (Table S7).

In conclusion, we propose that the levels of glutathione oxidation in CSF could serve as a stratification biomarker to select ALS patients for antioxidant therapy and an approach to monitoring treatment responses to therapeutic agents targeting oxidative stress. Future studies with larger, independent cohorts of patients are needed to replicate these findings. However, our results support further evaluation of tGSH and GSSG in the setting of therapeutic intervention in clinical trials of therapies targeting oxidative stress.

## Supplementary Information

Additional file 1: **Materials and Methods**. **Fig. S1** MS/MS fragments of GS-NEM and GS*-NEM, and p-MRM chromatography of GS-NEM and GS*-NEM co-eluted from HPLC. **Fig. S2** Chemical structures of selected ions derived from the fragmentation of GS-NEM and GS* NEM. **Table S1** Subject demographics and clinical details. **Table S2** Proteins with significantly altered abundance in ALS compared to HC groups in two consecutive visits. **Table S5** Mean concentrations of GSH, tGSH and GSSG in CSF of ALS patients and HC volunteers. Supplementary Discussion.

Additional file 2: **Table S3**. Dataset of proteins identified and quantified in CSF samples from ALS patients and healthy controls

Additional file 3: **Table S4**. Dataset of peptides containing Cysteine (C) residues derivatised by IAM or NEM identified and quantified in CSF samples from ALS patients and healthy controls

Additional file 4: **Table S6**. LLOD and LLOQ of GSH determined by various approaches and chemicals used for derivatisation.

Additional file 5: **Table S7**. Regression models displaying correlations between potential biomarkers and clinical parameters.

## Data Availability

The mass spectrometry data have been deposited to the ProteomeXchange Consortium via the PRIDE partner repository with the dataset identifiers PXD053913 and PXD053927.

## Abbreviations

ALSFRS-R: Revised amyotrophic lateral sclerosis functional rating scale
CSF: Cerebrospinal fluid
Cys: Cysteine
GSH: Reduced glutathione
GSH*: Glycine-^13^C_2_, ^15^N-Glutathione
tGSH: Total glutathione
GS-NEM: Derivatisation of GSH by NEM
tGS-NEM: Derivatisation of tGSH by NEM
GSSG: Oxidised glutathione
HC: Healthy control group
HPLC: High-pressure liquid chromatography
ALS: Amyotrophic lateral sclerosis
MRM: Multiple reaction monitoring
MS: Mass spectrometry
NEM: N-Ethylmaleimide
TCEP: Tris(2-carboxyethyl)phosphine
