# Supplementary material for: Glutathione oxidation in cerebrospinal fluid as a biomarker of oxidative stress in amyotrophic lateral sclerosis
Source: Transl Neurodegener. 2025 Jul 7;14:36. doi: 10.1186/s40035-025-00496-3 (PMC12232736; doi:10.1186/s40035-025-00496-3)
Supplement: Supplementary file 1 — Additional file 1: Materials and Methods. Fig. S1 MS/MS fragments of GS-NEM and GS*-NEM, and p-MRM chromatography of GS-NEM and GS*-NEM co-eluted from HPLC. Fig. S2 Chemical structures of selected ions derived from the fragmentation of GS-NEM and GS* NEM. Table S1 Subject demographics and clinical details. Table S2 Proteins with significantly altered abundance in ALS compared to HC groups in two consecutive visits. Table S5 Mean concentrations of GSH, tGSH and GSSG in CSF of ALS patients and HC volunteers. Supplementary Discussion. [file 40035_2025_496_MOESM1_ESM.pdf]

Supplementary Information

Materials and Methods

Chemicals

Iodoacetamide (IAM), Dithiothreitol (DTT) and N-Ethylmaleimide (NEM) (Merck, UK) were dissolved in water to make a stock of 0.25 M, while TCEP 0.5M (Merck, UK) was used as a stock. Both GSH and GSSG were purchased from Merck, while heavy isotope labelled GSH (glycine-13C2, 15N-Glutathione) (GSH*) was purchased from Cambridge Isotope (UK). All of these reagents were resuspended in water to make a stock of 1 mg/mL for each and used as standards and internal standards (GSH*). HPLC-MS grade water, trifluoroacetic acid (TFA), formic acid (FA), acetonitrile (ACN) and 3kDa Amicon ultra-0.5 centrifugal filter unit were also purchased from Merck (UK), while spinning C18 cartridges were purchased from Thermo (UK).

CSF samples

Samples were collected from participants enrolled in the observational 'NRF2 Biomarkers' study. The study was approved by an NHS Research Ethics committee (reference: 18/Y.H./0253). Patients were recruited at the Royal Hallamshire Hospital, Sheffield, UK, and provided written informed consent. A lumbar puncture was performed, together with clinical assessments and characterisation of the disease state and rate of disease progression. CSF samples were immediately placed on ice and centrifuged at 2350g for 10 min at 4°C within 1 h of sampling and then transferred to a -80°c freezer. They were subsequently stored in liquid nitrogen. Demographic and clinical details of the subjects are shown in **Table S1**.

Protein sample preparation and immunodepletion

A protease inhibitor cocktail (leupeptin 1 µM, bestatin 130 µM and aprotinin 0.15 µM, all in working concentration) was added to 800 µL of CSF. Samples were concentrated using amicon ultra-15 centrifugal filter units (Millipore) with a 3 kDa molecular weight cut off (MWCO) at 4°C for 90 min at 4,000 x g. After centrifugation, the concentrated protein (~ 200 µL) was collected and transferred to a new 1.5 mL Eppendorf tube. The flow-through fraction (< 3 kDa) was collected and used for glutathione measurement described below. Concentrated protein samples were then immunodepleted using a multi-affinity removal spin cartridge human-14 (0.45 mL, Agilent Technologies, UK) to remove high-abundance proteins according to the manufacturer's protocol with some modifications. Briefly, 200 µL of buffer A was added to the concentrated CSF sample and incubated in the cartridge at room temperature for 5 minutes before centrifuging at 100 x g for 1.5 minutes. The supernatant containing low abundance proteins was collected into a new clean 2 mL Eppendorf tube. Another 400 µL of buffer A was added into the cartridge to elute unbound (low

abundance) proteins. The supernatant was then collected by centrifugation at room temperature for 3 min and then combined with the previous supernatant. The combined low abundance protein fraction was transferred to another centrifugal filter unit (3kDa MWCO) and centrifuged at 4°C for 90 min at 4,000 x g to concentrate proteins. Subsequently, the concentrated proteins (~200 µL) inside the filter unit were transferred to a new 2 mL Eppendorf tube. Then 1mL of (-20°C) ice-cold acetone was added into the tube and left at -20°C overnight to precipitate protein. The pellet of precipitated protein was collected by centrifugation at 21,000 x g for 15 min at 4°C. Acetone was then discarded, and the pellet was left to dry before 25 µL of protein extraction buffer (5% sodium dodecyl sulfate (SDS) in 50 mM triethylammonium bicarbonate TEAB pH 7.1 was added to dissolve the protein pellet. A volume of 4 µL was used for total protein concentration determination using a microBCA assay (Thermo, UK). 25 µg of protein from each sample was then digested in a suspension trapping device (S-trap) (Protifi, USA). Proteins were firstly alkylated using 15 mM iodoacetamide (IAM), shaking at 850 rpm in a thermomixer (Eppendorf, UK) in the dark for 45 min at room temperature, and then reduced using 35 mM DTT with the same previous condition before subjecting to a second alkylation by 60 mM NEM using the same conditions. Subsequently, samples were centrifuged at 21,000 x g for 15 min before transferring to an S-Trap microcolumn (≤ 100 µg) (Protifi, USA) for protein digestion according to the manufacturer's protocol. Briefly, proteins were trapped twice and washed 3 times with 100 mM TEAB pH 7.5 in 90% methanol before digestion in the S-trap using trypsin (Pierce, UK) (1:10 ratio of trypsin: protein) at 37°C overnight. Peptides were subsequently eluted with (60 µL each) 50 mM TEAB pH 8.5, and 0.2% formic acid (FA) then twice with 0.5% FA in 50% acetonitrile. The eluted peptides were dried in a vacuum concentrator (Eppendorf, UK) and resuspended in 50 µL of 0.5% FA for mass spectrometry analysis (MS).

GSH preparation, reduction and alkylation

A workflow for processing and measuring glutathione in CSF is shown in **Fig. 1b**. For measurement of GSH in CSF, 300 µL of flow-through from the initial amicon concentration step above (< 3 kDa MWCO) was derivatised (alkylated) with NEM 20 mM and incubated in a thermomixer (Eppendorf, UK) at 25°C for 45 min at 850 rpm in the dark. For tGSH measurement, another 300 µL of flow-through CSF was first reduced using 10 mM TCEP at 25°C for 45 min at 850 rpm and then derivatised with 20 mM NEM. To clean up derivatised samples (GS-NEM), all samples were subjected to solid-phase extraction using C18 cartridges. TFA was added to the derivatised samples to reach a final concentration of 0.1%. The samples were then centrifuged at 21,000 x g for 15 min; supernatants were collected for desalting, which was performed according to the manufacturer's protocol with some modifications. Briefly, the binding step was performed twice to maximise the binding of GS-NEM to C18 material; the C18

columns were washed three times with 300 µL of 0.1% TFA and then eluted with 25% ACN in 0.1% TFA and collected into a 2 mL Eppendorf tube. All samples were then dried in a vacuum concentrator (Eppendorf, UK) and stored at -80°C for MS-based analysis. Both light (GSH) and heavy stable isotopes (GSH*) of glutathione were also derivatised with NEM in the same way described for the CSF samples. Furthermore, to evaluate both the efficiency of the reduction step and the loss of GS-NEM during the C18 cleaning step, a known amount of SGGG (1.3 µM) was used for reducing, alkylating and subjecting to the desalting step and treated the same way compared to CSF samples.

GSH standard curve and MS performance evaluation

A linear standard curve of GS-NEM, in which 4 µL of 1.3 µM GS*-NEM was added into 38 µL of 0.5% FA for each GS-NEM concentration to serve as an internal standard. The standard curve was performed and established with concentrations of GS-NEM consisting of 0.13, 0.33, 0.65, 1.30, 2.60, 6.51, 13.02 and 26.03 µM. Triplicate measurements were made for each GS-NEM concentration. Quality control during MS analysis was also performed using the internal GS*-NEM standard to check the accuracy of glutathione measurements and the MS performance. A volume of 18 µL, in which every 4 µL of GS*-NEM (1.3 µM) was added to 38 µL of 0.5% FA, was run on an Orbitrap Elite (Thermo, UK) every 24h. The intensities of these quality control samples were then analysed to evaluate the performance of the MS instrument. Furthermore, a blank was also run after every 6 CSF samples to monitor for potential chromatography contamination. The recovery of GSH during sample preparation was assessed by spiking 50 fmol of GSH into CSF samples (n = 3) before sample preparation, and CSF control samples (without the addition of GSH) (n = 3) were also performed. The recovery of GSH was calculated as follows:

Recovery (%) = (Amount of measured GSH amount in spiked sample-amount of measured GSH in control sample)/(Theoretical amount of spiked GSH) X 100

Nano-HPLC and MS analysis

For Oxi-proteomics analysis

18 µL of resuspended peptides was injected and analysed by nanoflow LC-MS/MS using an Orbitrap Elite hybrid mass spectrometer (Thermo, UK) equipped with a nanospray source, coupled to an Ultimate RSLCnano LC System (Dionex, UK) and Tune Plus for Orbitrap Elite (Thermo). Peptides were desalted on-line using a nano C18 trap column, 75 µm ID x 20 mm (Thermo) and separated using a 130-min gradient starting from 3 to 40% buffer B consisting of 0.5% FA in 80% ACN on an EASY-Spray column, 50 cm × 50 µm ID, PepMap C18, 2 µm particles, 100 Å pore size (Thermo). The Orbitrap Elite was operated with a cycle of one MS (in the Orbitrap) acquired at a resolution of 120,000 at m/z 400,

with the top 20 most abundant multiply charged (2+ and higher) ions in a given chromatographic window subjected to MS/MS fragmentation in the linear ion trap. An FTMS target value of $1e^6$ and an ion trap MSn target value of $1e^4$ were used with the lock mass (445.120025) enabled. Maximum FTMS scan accumulation time of 200 ms and maximum ion trap MSn scan accumulation time of 50 ms were used. Dynamic exclusion was enabled with a repeat duration of 45 s with an exclusion list of 500 and an exclusion duration of 30.

GSH MS analysis

The dried derivatised CSF samples were resuspended in 38 µL of 0.5% FA, and 4 µL of GS*-NEM (1.3 µM) was then added to serve as an internal standard. 18 µL of each sample was injected and analysed by a nano-HPLC (Dionex, UK) coupled to an Orbitrap Elite (Thermo, UK). Derivatised compounds were separated using a PepMap RSLC C18 column (2 µm, 100 Å, 50 µm x 15 cm) (Thermo, UK) operated at 40oC at a flow of 0.25 µL /min during a 35 min gradient generated by a solvent A consisting of 0.1% FA in water and solvent B containing 0.1% FA in 80% ACN. The gradient was run at 3% of solvent B for 10 min, then ramped up to 45% solvent B over 15 min and a second ramp to 90% solvent B over 2 min, isocratic run at 90% solvent B for 5 min, then decreased to 3% B for 5 min. The MS was activated 10 minutes after starting the HPLC and acquired data over 25 minutes. Both GS-NEM and GS*-NEM co-eluted at retention time (Rt) 19 min (on HPLC chromatography) or appeared at Rt = 9 min (on MS chromatography). GS-NEM, GS*-NEM and other analytes in each CSF sample were ionised using electrospray ionisation (ESI) operated at 50°C, sheath gas 6, and spray voltage 4000 V, with a capillary temperature of 300°C. The Orbitrap Elite MS was operated in positive ion trap mode with a collision energy of 35. For quantitative analysis of both GS-NEM and GS*-NEM, the following transitions were monitored using pseudo-MRM (multiple reaction monitoring) as follows: for GS-NEM (m/z): 433 → 304 and 433 → 287, for GS*-NEM (glycine-13C2, 15N-GS-NEM) (m/z): 436 → 307 and 436 → 290. Furthermore, un-derivatised GSH and intact GSSG forms were also monitored to ensure that both reduction and alkylation processes were complete. Therefore, transitions (m/z) of 308 → 179 and 308 → 162 (for monitoring GSH), and 307 → 299, 307 → 276, 613 → 484 and 613 → 355 (for monitoring GSSG in $[M+2H]^{2+}$ and $[M+H]^{1+}$ forms respectively). However, neither underivatised GSH or GSSG were detected in CSF samples. MS data were acquired with Xcalibur V 3.0.63 software (Thermo, UK).

Data analysis

For Oxiproteomics analysis

Raw MS data were processed with MaxQuant V.1.6.10.43, searching against a human UniProt sequence database downloaded from https://www.uniprot.org/ (Mar 2021) using the following

search parameters: Trypsin/P for digestion with 2 missed cleavages, methionine oxidation (M), N-terminal protein acetylation, and Carboxyamidomethylation (CAM, 57 Da) and N-ethylmaleimide (NEM, 125 Da) on cysteine were set as variable modifications. The first and main searches were carried out with MS tolerance of 10 and 5 ppm, respectively. Label-free Quantification (LFQ) was enabled with a minimum ratio count of 2, a minimum number of neighbours of 3 and an average number of neighbours of 6. PSM and protein match thresholds were set at 0.1 ppm. A protein false discovery rate (FDR) of 0.01 and a peptide FDR of 0.01 were used for identification level cut-offs. LFQ was performed using only peptides containing no PTM (except for acetyl protein N-term). Peptides containing cysteine modified by CAM was considered as initially reduced Cys-containing peptides while peptides containing NEM modification on cysteine were oxidised Cys-containing peptides. The levels of both reduced and oxidised Cys-containing peptides were used to determine the levels of cysteine oxidation in ALS and HC groups. Changes in cysteine oxidation levels were normalised to protein expression determined by LFQ intensities.

The peptide and protein data from Maxquant outputs were further analysed using Perseus software V.1.6.1.50. The ProteinGroup.txt file was used for LFQ, while IAMPeptide.txt and NEMPeptide.txt files were used for cysteine oxidation calculations. The reversed and potential contaminant proteins/peptides were removed before analysis. For Cys-containing peptide analysis, an expand site table step was performed before any analysis was carried out. The intensity of proteins/peptides was transformed to log2 before further analysis. At least 70% valid value for one of the groups was used for data filtering, proteins in each sample were normalised to median and missing values were imputed using normal distribution with width of 0.3 and down shift of 1.8. Finally, multiple ANOVA tests were performed to investigate differently abundant proteins at an FDR of 0.05 in ALS vs HC comparisons as well as between two visits to examine their association with disease progression.

The lists of differently significant expressed (oxi)proteins (**Table S4**) were used for the determination of correlations with clinical parameters using a simple regression model performed by Graphpad.

For GSH analysis

MS data were analysed using Skyline version 21.1.0.218 using the small molecule analysis approach to obtain values of peak areas, and ratios of light (GS-NEM) and heavy (GS*-NEM) derivatised compounds. Ratios of light/heavy GS-NEM were referred to the standard curve to obtain concentrations of reduced and total reduced GSH. The obtained concentrations were then multiplied by a dilution factor of 7.14 (~ 40 µL /300 µL) to calculate concentrations of GSH and tGSH in CSF. The concentration of GSSG was calculated based on a subtraction of tGSH and GSH concentrations.

Extracted data were exported into data spreadsheets, and further statistical analyses were carried out using Excel 2016 (Microsoft, USA) and GraphPad Prism V 9.2.0 (GraphPad Software, USA).

Welch's t-test was performed for each comparison to determine if any change of glutathione in CSF occurred between ALS and HC groups for 1st and 2nd visits. Using GraphPad, two-tailed Pearson correlation and a simple linear regression model were utilised to identify correlations between clinical parameters and glutathione in the CSF from ALS patients.

Development of targeted MS-based glutathione oxidation assay

Before pseudo-MRM experiments were designed, full MS/MS scans of GS-NEM and GS*-NEM were acquired on an Orbitrap Elite system with a collision energy of 35 eV to obtain the MS/MS fragment profile, as shown in **Figs. S1 A**-**B**. Of these fragment ions, only the highest abundance ions containing glycine-13C2, 15N were selected for the pseudo-MRM experiments. These signature ions were also annotated with chemical structures of GS-NEM and GS*-NEM, as shown in **Figs. S1 A-B**. In LC-MS analysis, both GS-NEM and GS*-NEM co-eluted in a single peak (**Fig. S1**). GS-NEM (433 m/z) and GS*-NEM (436 m/z) ions were fragmented by collision-induced dissociation (CID), and selected fragment ions of 304 and 287 m/z were monitored in pairs with their corresponding parent ion 433 m/z for GS-NEM, and fragment ions of 307 and 290 m/z were monitored for internal standard ion 436 m/z (GS*-NEM) (**Fig. S2**). Other pairs of ions were also monitored to ensure that alkylation and reduction reactions were completed. There were no other forms of GSSG and GSH detected, indicating that the alkylating and reducing steps were complete. A linear coefficient of 0.9969 was obtained for the GS-NEM standard curve, corresponding to GSH concentrations ranging from 0.13 to 26 µM (**Fig. 1c**). A total of 140 CSF samples were analysed using this MS method, and internal standards were run after every 20 samples to evaluate the performance of the nano-HPLC MS/MS system. As a result, a coefficient variation (CV) of 5.0% was obtained for the internal standards alone (**Fig. 1d**), suggesting a good performance of the instrument system during MS runs.

Signals of these internal standards were extracted and plotted in **Fig. 1e** for two separate batches, GS-NEM and tGS-NEM measurements. CVs of internal standards in both batches were similar at 13.13 and 13.57% for reduced and oxidised measurements, respectively. The lower limit of detection (LLOD) and lower limit of quantitation (LLOQ) of GS-NEM (and also for tGS-NEM) were 0.04 and 0.13 µM, respectively, which were ten times lower than previously reported values [21] (0.4 µM and 1.5 µM for LLOD and LLOQ respectively). This suggests that our optimised MRM assay gives better detection sensitivity for the quantitation of glutathione in human CSF. **Table S6** shows the LLOD and LLOQ values in this study compared to other published values obtained by different approaches in various

biological samples. Furthermore, CVs of 3.75 %, 4.91% and 5.1% were also determined for intraday, interday and accuracy for recovery tests, respectively.

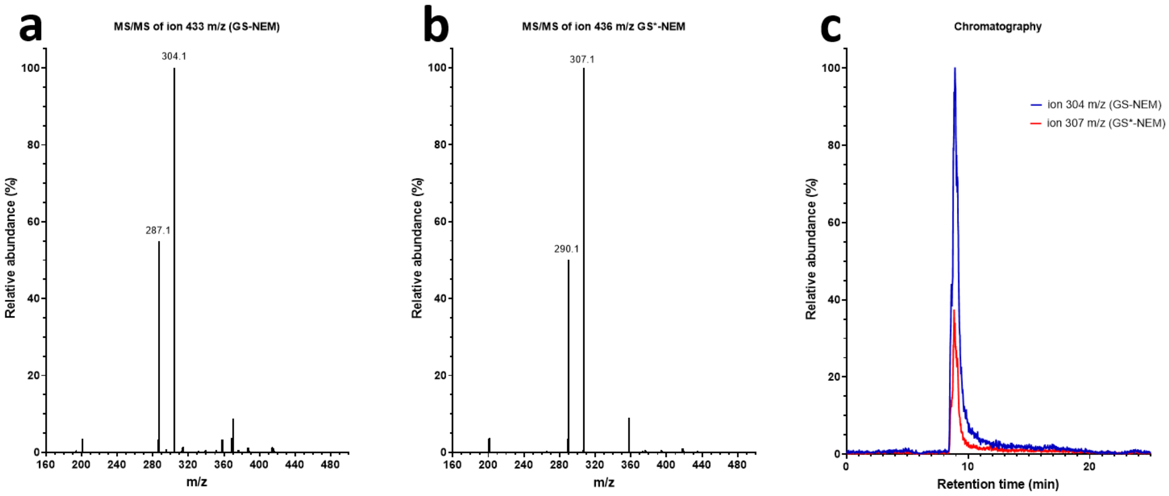

Fig. S1. MS/MS fragments of GS-NEM (A) and GS*-NEM (B), and p-MRM chromatography of GS-NEM and GS*-NEM co-eluted from HPLC (C). Structures of selected ions used for p-MRM experiments are shown in Figs. S2. A and B.

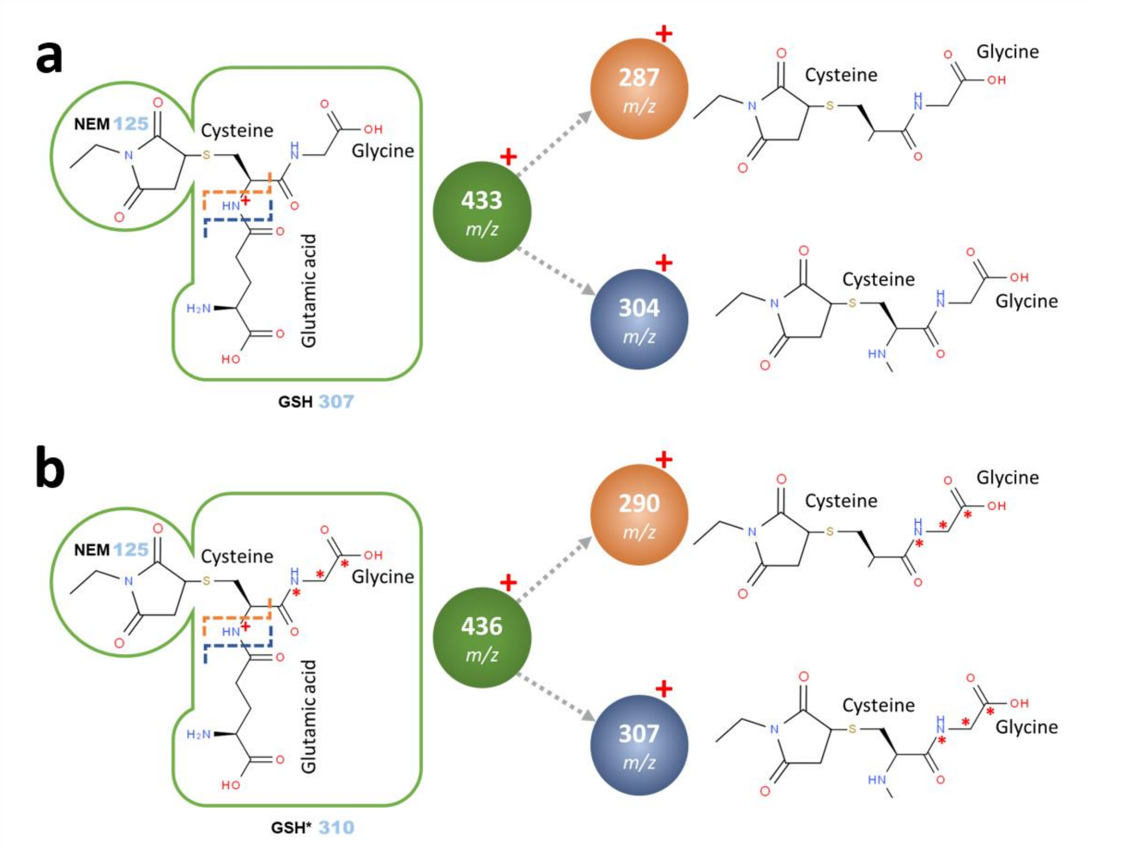

Fig. S2. Chemical structures of selected ions derived from the fragmentation of GS-NEM and GS*-NEM.

Supplementary Table S1. Subject demographics and clinical details.

		ALS visit 1	ALS visit 2	Healthy Controls
		(n = 24)	(n = 15)	Visit 1 (n = 20) Visit 2 (n = 11)
**Gender**	Male, *n* (%)	19 (68%)	8 (47%)	7 (35%)
**Age (years)**	Mean (range)	63 (36-82)	59 (36-70)	59 (36-80)
**Site of onset**	Limb	26	13	-
	Bulbar	4	4	-
**ALSFRS-R**	Mean (range)	38.2 (27-47)	39.3 (31-44)	-
**Disease progression rate[1]**	Mean (range)	0.9 (0.1-6.0)	0.4 (0.1-1.0)	-
**Disease duration[2] (months)**	Mean (range)	18.8 (3-79)	26.5 (11-83)	-

[1]Disease progression rate = (48-ALSFRS-R)/disease duration (months). [2]Disease duration = time from symptom onset until lumbar puncture.

Supplementary Table S2. Proteins with significantly altered abundance in ALS compared to HC groups in two consecutive visits.

Gene	AC	Name	Log2 T-test difference 1st Visit	p Value 1st Visit	Log2 T-test difference 2nd Visit	p Value 2nd Visit
CHI3L2	Q15782	Chitinase-3-like protein 2	**1.49**	**1.20E-08**	**1.26**	**8.85E-06**
CHI3L1	P36222	Chitinase-3-like protein 1	**0.70**	**1.80E-07**	**0.55**	**9.72E-04**
SEPINA3	P01011	Alpha-1-antichymotrypsin	**0.80**	**6.80E-05**	0.31	5.95E-02
CHIT1	Q13231	Chitotriosidase-1	**2.88**	**1.30E-04**	**2.92**	**1.55E-03**
FCGR3A	P08637	Low affinity immunoglobulin gamma Fc region receptor III-A	**0.52**	**1.70E-04**	**0.54**	**1.78E-02**
C7	P10643	Complement component C7	**0.38**	**3.60E-04**	0.14	2.51E-01
MST1	P26927	Hepatocyte growth factor-like protein	**0.83**	**3.60E-04**	0.26	2.88E-01
CFD	P00746	Complement factor D	**0.54**	**8.30E-04**	0.08	5.54E-01
CFH	P08603	Complement factor H	**0.38**	**1.50E-03**	0.06	6.90E-01
C3	P01024	Complement C3	**0.54**	**1.80E-03**	-0.01	9.53E-01
MASP1	P48740	Mannan-binding lectin serine protease 1	**0.52**	**2.60E-03**	0.20	2.30E-01
SERPING1	P05155	Plasma protease C1 inhibitor	**0.35**	**2.80E-03**	-0.01	9.46E-01
MAN1B1	Q9UKM7	Endoplasmic reticulum mannosyl-oligosaccharide 1,2-alpha-mannosidase	-0.38	2.90E-03	**0.18**	**5.85E-01**
COL18A1	P39060	Collagen alpha-1(XVIII) chain	**0.36**	**4.80E-03**	**0.29**	**5.22E-02**
NPC2	P61916	NPC intracellular cholesterol transporter 2	0.62	7.10E-03	**0.69**	**1.73E-01**
EFEMP1	Q12805	EGF-containing fibulin-like extracellular matrix protein 1	**0.52**	**1.60E-02**	0.45	1.23E-02
DMD	A0A087WV90	Dystrophin	1.85	1.10E-01	**4.79**	**4.45E-04**
VASN	Q6EMK4	Vasorin	0.17	1.30E-01	**0.37**	**8.41E-04**
SNED1	Q8TER0	Sushi, nidogen and EGF-like domain-containing protein 1	0.19	1.40E-01	**0.41**	**1.01E-02**
CBR1	P16152	Carbonyl reductase [NADPH] 1	-0.29	2.70E-01	**0.61**	**7.62E-02**
CA11	O75493	Carbonic anhydrase-related protein 11	0.38	3.00E-01	**0.91**	**2.49E-02**
VGF	O15240	Neurosecretory protein VGF	-0.42	3.20E-01	**1.36**	**2.47E-03**
PKM	P14618	Pyruvate kinase PKM	-0.09	4.60E-01	**0.78**	**2.69E-02**
GALNT10	Q86SR1	Polypeptide N-acetyl-galactosaminyltransferase 10	0.16	5.50E-01	**1.19**	**3.63E-04**
PGK1	P00558	Phosphoglycerate kinase 1	0.06	6.30E-01	**0.63**	**3.04E-02**
ECM2	O94769	Extracellular matrix protein 2	0.03	8.40E-01	**0.28**	**2.27E-01**
PRG4	Q92954	Proteoglycan 4	-0.01	9.80E-01	**1.33**	**2.73E-03**
MAN2A1	Q16706	Alpha-mannosidase 2	0.00	9.90E-01	**0.55**	**7.50E-03**

Bold values indicate statistically significant (Multiple sample ANOVA test) proteins in ALS vs HC (FDR 0.05). The full dataset is available in Supplementary Table S3.

Supplementary Table S5. Mean concentrations of GSH, tGSH and GSSG in CSF of ALS patients and HC volunteers.

Glutathione	HC		ALS	
	1st visit	2nd visit	1st visit	2nd visit
tGSH (µM)	0.33 ± 0.08	0.28 ± 0.08	0.44 ± 0.2	0.43 ± 0.16
GSH (µM)	0.09 ± 0.05	0.09 ± 0.04	0.08 ± 0.03	0.07 ± 0.02
GSSG (µM)	0.12 ± 0.05	0.09 ± 0.04	0.18 ± 0.1	0.18 ± 0.08
GSSG/GSH (fold)	1.60 ± 0.94	1.33 ± 1.03	2.88 ± 2.75	2.84 ± 1.95

Welch's t-test was performed to identify significant differences between the groups. Individual data points are plotted in Figs. 1f-i.

Supplementary Discussion.

This study aimed to develop an approach to measure biomarkers of disease progression relevant to oxidative stress that could be used for target engagement studies in clinical trials. Therefore, we developed a workflow using MS-based approaches to measure protein abundance, cysteine oxidation, and the abundance and oxidation state of glutathione in CSF from a cohort of patients with ALS and a group of healthy controls at two time points. To reduce the very high dynamic range of protein abundance in CSF, we immuno-depleted samples to remove the top 14 most abundant proteins. This enhanced the proteome coverage up to 3.5-fold compared to non-depleted CSF (data not shown). As a result, 1,561 proteins were detected in the entire data set, and 699 proteins were quantified in 70% of replicates (for each group, HC 1st, HC 2nd, ALS 1st, ALS 2nd).

We identified several proteins with significantly increased abundance in ALS compared to the HC group in both consecutive visits. These included chitinase-3-like protein 1 and 2 (CHI3L1 and CHI3L2), chitotriosidase-1 (CHIT1) and collagen alpha-1(XVIII) chain (COL18A1), and our data confirm these proteins as candidate biomarkers of ALS as previously reported [1] [2]. So far, CHI3L1 and CHIT1 in CSF have been identified as potential biomarkers in Alzheimer's disease (AD), multiple sclerosis (MS) and other neurological diseases [3]. The levels of CHIT1, CHI3L1 and CHI3L2 in CSF of ALS patients were reported to be elevated compared to HC groups (using ELISA) [4, 5]. Furthermore, CHIT1 and CHI3L2 correlated with disease progression rate, and CHI3L1 correlated with the degree of cognitive

dysfunction. CHIT1 levels were also associated with survival in multivariate models, and chitinase levels were longitudinally stable [4, 5]. However, in our data, the abundance of CHIT1, CHI3L1, CHI3L2, and collagen alpha-1(XVIII) chain did not change between the two visits in the ALS group, indicating that their expression level was stable as the disease progressed.

Protein alpha-1-antichymotrypsin (SERPINA3) was significantly elevated in ALS patients (compared to the HC group) at the first visit, suggesting it could play a potential role in ALS development since it positively and negatively correlated with disease progression rate and ALSFRS-R score, respectively. Moreover, the increased expression of alpha-1-antichymotrypsin together with CFH, C7, MASP1, SERPING1, FCGR3A, CFD, CHI3L1 and CHI3L2 might involve the immune system response and response to stress as an imbalance of the immune response could contribute to excessive inflammation occurring early during disease progression [6].

In terms of disease progression, there was a significant positive correlation between the increase in protein CA11 expression and the increase in disease progression rate over time (4 months apart). This means that the increase in progression rate from the first to the second visit is positively correlated with the increased abundance of protein C11 between these time points in ALS patients. The protein CA11 is a member of the carbonyl reductase family of enzymes, which play a role in the reduction of various carbonyl compounds, including reactive oxygen species (ROS) and oxidative stress. This protein has a potential role in neuroprotection as it is essential for neuronal cell survival and protection against oxidative stress [7]. Additionally, the carbonic anhydrase family has been shown to have a role in the regulation of the immune system, including the activation of microglia [8].

We next investigated if any oxidative biomarkers could be identified in the CSF of ALS patients and found that several proteins exhibited significantly altered cysteine oxidation in ALS versus the HC group (**Table S4**). Brevican core protein (BCAN), exhibited increased (reversible) oxidation at Cys688 and Cys699 in ALS vs HC groups in both consecutive visits. This protein is a CNS-specific extracellular matrix proteoglycan and is degraded by extracellular metalloproteinases, proposing an unknown transport mechanism from the brain parenchyma into CSF [9]. Protein dipeptidyl peptise 2 DPP7 contained two redox cysteine sites (Cys332 and Cys338) more oxidised in ALS than HC for both visits. This protein is a member of the serine peptidase and plays a key role in the degradation of oligopeptides [10]. Proline-specific dipeptidyl peptidases (DPPs) have emerged as targets for drug development. Cys332 and Cys382 form a disulphide bond in the loop insertion, defining dipeptidyl aminopeptidase specificity and acting to stabilise the long loop [11]. Our data could indicate that more disulphide bonding occurs in ALS patients than in the HC group. Although 17 cysteines (from 14 different proteins) were irreversibly oxidised (O2 and O3), none of these were significantly altered in

abundance in ALS compared to HC groups, but some proteins increased their irreversible oxidation in both ALS and HC between the first and second visits (**Table S4**).

We have identified a set of proteins with significantly altered abundance and/or oxidation in ALS compared to healthy control groups. These proteins are involved in key pathological processes such as neuroinflammation, extracellular matrix remodeling, metabolic dysregulation, synaptic dysfunction, and impaired proteostasis. For example, CHI3L2 and CHIT1 are involved in the degradation of chitin-like substrates, and their elevated abundance suggests an activated microglial response and ongoing neuroinflammation in ALS [1-4]. Complement components C3, C5, and C9 are involved in the activation of the complement cascade, contributing to neuroinflammatory damage [12, 13]. SERPINA3, an acute-phase protein, is upregulated during inflammatory responses and is implicated in neurodegenerative processes [6]. Metabolic enzymes PKM and PGK1 play important roles in glycolysis, and their increased expression may reflect metabolic adaptations or dysfunctions in ALS-affected neurons [14]. Proteins involved in extracellular matrix organisation, such as FN1 and LGALS3BP, their high abundance suggested alterations in cell adhesion and signalling pathways, and protein VASN might modulate TGF-beta signalling, and disruptions in neuroprotective signalling mechanisms [15]. Elevated oxidation of BCAN and CNTNAP2 might be involved in disease-specific extracellular matrix remodelling and neuronal connectivity disruptions in ALS, respectively [9, 16]. DPP7, a lysosomal protease, its function might not directly link to ALS, but it might be involved in protein catabolism and turnover, processes that are often disrupted in neurodegenerative diseases [10]. PTGDS, which catalyses the conversion of prostaglandin H2 to prostaglandin D2, was implicated in modulating neuroinflammation and may influence glial activation in ALS [17]. We next tested whether the level of glutathione or its oxidation state was altered in ALS versus healthy controls and between consecutive visits in the ALS cohort. The LC-MRM approach has been widely used in targeted metabolomics and proteomics analyses because of its high specificity, selectivity, accuracy, precision and robustness. Several MRM methods were successfully developed to measure glutathione levels in blood [18] and other biological samples using different MS-based approaches [19-21] (**Table S6**). However, to our knowledge, there are no previous reports using this approach to measure glutathione in the CSF, which has much lower levels of glutathione compared to other biofluids (see **Table S6**) and, therefore requires the higher sensitivity afforded by nano-flow chromatography.

In our MRM method development process, we generated a GSH standard curve for calculating GSH and tGSH concentrations in human CSF. Compared with the GSH standard curves reported [18] and [22], the slope of our standard curve was consistent with their standard curves, 0.9038 in our study compared to the slope of 0.9177 and 0.932 in these reports, respectively. A linear coefficient R^2

= 0.9969 in our data was in line with R^2 = 0.999 and R^2 = 0.9986 found in [18] and [22], respectively. Furthermore, a linear dynamic range (0.13 - 26 µM) in our data is much lower than in these previous reports (25 - 500 µM and 8 - 256 µM as reported in [18] and [22], respectively). This allowed us to successfully measure the much lower glutathione levels in CSF compared to blood.

When measuring glutathione (GSH and GSSG) in clinical samples, it is important to inhibit the auto-oxidation of GSH (into the GSSG form), which could occur during sample collection and preparation. Therefore, we utilised NEM to block the sulfhydryl group on the cysteine residue of GSH via alkylation (to form derivatised GS-NEM) in order to prevent GSH auto-oxidation [23]. This also helps to prevent the enzymatic reduction of GSSG and inhibits glutathione reductase activity [23]. The concentrations of tGSH detected in the HC group (0.33 ± 0.08 and 0.28 ± 0.08 µM for the first and second visits, respectively) were similar to those previously reported in which tGSH was measured by a spectrophotometric approach [24]. It is noted that only healthy males participated (n = 26) in that study. The mean tGSH concentrations of 0.12 ± 0.02, 0.18 ± 0.01 and 0.14 ± 0.01 µM were reported for three different fractions 0–6, 7–12 and 13–18 mL, respectively [24], while our data from a male healthy volunteer group for the first and second visits were 0.31 ± 0.10 (n = 7) and 0.29 ± 0.09 µM (n =5), respectively. The concentrations measured in our study were approximately 40% higher than the values previously reported [24]. This difference could result from the different methodologies used. However, the difference, in our opinion, was not significant, and we considered that our data did not conflict with the levels reported in the literature. Subsequently, this allowed us to examine if we could use levels of glutathione and/or its oxidation as potential biomarkers of oxidative stress in ALS.

Oxidative stress plays a key role in the pathophysiology of ALS [25, 26], but most antioxidants, including glutathione, have failed to slow ALS progression in clinical trials [27, 28]. Several potential biofluid-based biomarkers have been discovered for ALS [29-31], including neurofilament light chain (NfL), which is considered the best-performing candidate for therapeutic trials [32]. Unfortunately, neurofilament light chain was not detected in our data, potentially due to relatively low abundance in CSF or loss of the protein during the immunodepletion step. The failure to detect neurofilament light chain has been reported in other global proteomic studies of CSF from ALS patients [2] but has been successfully detected using immunoprecipitation–tandem mass spectrometry method in which peptides derived from the protein are specifically enriched from CSF after digestion, before MS analysis [33].

Our data show that three crucial parameters, including tGSH and GSSG concentrations in ALS, were significantly higher than those in the HC group at the first visit and tGSH and GSSG concentrations

and the GSSG/GSH ratio in ALS at the second visit (**Table 3** and **Figs. 4B** and **C**). Compared to the healthy control group, the mean value of each parameter (tGSH, GSSG, and GSSG/GSH ratio) was almost unchanged in the ALS group for the second compared to the first visits (4 months apart), suggesting that the levels of these molecules remained high and unchanged once ALS is established. Our findings support the proposal that either tGSH or GSSG could be used as potential biomarkers in ALS.

There were strong positive correlations between disease duration until the first sample date and tGSH, GSSG and GSSG/GSH ratio parameters in the CSF of ALS patients for disease duration until the first sample date vs tGSH, GSSG and GSSG/GSH ratio, respectively. This suggests an accumulation of oxidative stress biomarkers over time, though these glutathione derivatives were relatively stable over the 4-month interval between sample collections in this study. There was no difference in the concentrations of GSH, tGSH and GSSG between slow-, intermediate-, and fast-progressing ALS cases measured by the rate of change of the ALS-FRS-R score per month.

The high concentrations of tGSH, GSSS, and GSSG/GSH ratio, as well as total reversibly oxidised Cys content, reflected the imbalance between the antioxidant defence system and ROS production, contributing to the development and progression of motor neuron injury in patients with ALS [48]. The measurement of tGSG, GSH and corresponding GSSG/GSH ratio could be used as biomarkers in ALS as the GSSG/GSH ratio showed a strongly positive correlation with the level of Cys oxidation in ALS patients, and the longer the disease duration, the stronger was the correlation observed. A higher GSSG/GSH ratio was also reported in a SOD1-ALS cell model [34] and a SOD1-ALS mouse model [35].. Our data, especially the positive correlation between the protein Cys oxidation level and GSSG/GSH ratio, could offer potential biomarkers for the evaluation of oxidative stress mechanisms contributing to neurodegeneration in ALS and the effects of therapeutic interventions targeting oxidative stress.

Supplementary References

1. Bereman MS, Beri J, Enders JR, Nash T: **Machine learning reveals protein signatures in CSF and plasma fluids of clinical value for ALS.** *Scientific Reports* 2018, **8:**16334.
2. Dellar ER, Vendrell I, Talbot K, Kessler BM, Fischer R, Turner MR, Thompson AG: **Data-independent acquisition proteomics of cerebrospinal fluid implicates endoplasmic reticulum and inflammatory mechanisms in amyotrophic lateral sclerosis.** *Journal of Neurochemistry* 2024, **168:**115-127.
3. Kušnierová P, Zeman D, Hradílek P, Zapletalová O, Stejskal D: **Determination of chitinase 3-like 1 in cerebrospinal fluid in multiple sclerosis and other neurological diseases.** *PLOS ONE* 2020, **15:**e0233519.
4. Thompson AG, Gray E, Bampton A, Raciborska D, Talbot K, Turner MR: **CSF chitinase proteins in amyotrophic lateral sclerosis.** *Journal of Neurology, Neurosurgery & Psychiatry* 2019, **90:**1215-1220.
5. Gille B, De Schaepdryver M, Dedeene L, Goossens J, Claeys KG, Van Den Bosch L, Tournoy J, Van Damme P, Poesen K: **Inflammatory markers in cerebrospinal fluid: independent prognostic biomarkers in amyotrophic lateral sclerosis?** *Journal of Neurology, Neurosurgery & Psychiatry* 2019, **90:**1338-1346.
6. Béland LC, Markovinovic A, Jakovac H, De Marchi F, Bilic E, Mazzini L, Kriz J, Munitic I: **Immunity in amyotrophic lateral sclerosis: blurred lines between excessive inflammation and inefficient immune responses.** *Brain Commun* 2020, **2:**fcaa124.
7. Rashid MA, Haque M, Akbar M: **Detoxification of Carbonyl Compounds by Carbonyl Reductase in Neurodegeneration.** *Adv Neurobiol* 2016, **12:**355-365.
8. Liu X, Lu D-y, Bowser RP, Liu J: **Expression of Carbonic Anhydrase I in Motor Neurons and Alterations in ALS.** *International Journal of Molecular Sciences* 2016, **17**.
9. Hußler W, Höhn L, Stolz C, Vielhaber S, Garz C, Schmitt FC, Gundelfinger ED, Schreiber S, Seidenbecher CI: **Brevican and Neurocan Cleavage Products in the Cerebrospinal Fluid - Differential Occurrence in ALS, Epilepsy and Small Vessel Disease.** *Frontiers in Cellular Neuroscience* 2022, **16**.
10. Wagner L, Klemann C, Stephan M, von Hörsten S: **Unravelling the immunological roles of dipeptidyl peptidase 4 (DPP4) activity and/or structure homologue (DASH) proteins.** *Clin Exp Immunol* 2016, **184:**265-283.
11. Bezerra GA, Dobrovetsky E, Dong A, Seitova A, Crombett L, Shewchuk LM, Hassell AM, Sweitzer SM, Sweitzer TD, McDevitt PJ, et al: **Structures of Human DPP7 Reveal the Molecular Basis of Specific Inhibition and the Architectural Diversity of Proline-Specific Peptidases.** *PLOS ONE* 2012, **7:**e43019.
12. Hong S, Beja-Glasser VF, Nfonoyim BM, Frouin A, Li S, Ramakrishnan S, Merry KM, Shi Q, Rosenthal A, Barres BA, et al: **Complement and microglia mediate early synapse loss in Alzheimer mouse models.** *Science* 2016, **352:**712-716.
13. Ma SX, Seo BA, Kim D, Xiong Y, Kwon SH, Brahmachari S, Kim S, Kam TI, Nirujogi RS, Kwon SH, et al: **Complement and Coagulation Cascades are Potentially Involved in Dopaminergic Neurodegeneration in alpha-Synuclein-Based Mouse Models of Parkinson's Disease.** *J Proteome Res* 2021, **20:**3428-3443.
14. Manzo E, Lorenzini I, Barrameda D, O'Conner AG, Barrows JM, Starr A, Kovalik T, Rabichow BE, Lehmkuhl EM, Shreiner DD, et al: **Glycolysis upregulation is neuroprotective as a compensatory mechanism in ALS.** *Elife* 2019, **8**.
15. Costa J, Pronto-Laborinho A, Pinto S, Gromicho M, Bonucci S, Tranfield E, Correia C, Alexandre BM, de Carvalho M: **Investigating LGALS3BP/90 K glycoprotein in the cerebrospinal fluid of patients with neurological diseases.** *Sci Rep* 2020, **10:**5649.
16. Martin-de-Saavedra MD, Dos Santos M, Culotta L, Varea O, Spielman BP, Parnell E, Forrest MP, Gao R, Yoon S, McCoig E, et al: **Shed CNTNAP2 ectodomain is detectable in CSF and regulates Ca(2+) homeostasis and network synchrony via PMCA2/ATP2B2.** *Neuron* 2022, **110:**627-643 e629.
17. de Boer AS, Koszka K, Kiskinis E, Suzuki N, Davis-Dusenbery BN, Eggan K: **Genetic validation of a therapeutic target in a mouse model of ALS.** *Sci Transl Med* 2014, **6:**248ra104.
18. Moore T, Le A, Niemi AK, Kwan T, Cusmano-Ozog K, Enns GM, Cowan TM: **A new LC-MS/MS method for the clinical determination of reduced and oxidized glutathione from whole blood.** *J Chromatogr B Analyt Technol Biomed Life Sci* 2013, **929:**51-55.
19. Harwood DT, Kettle AJ, Brennan S, Winterbourn CC: **Simultaneous determination of reduced glutathione, glutathione disulphide and glutathione sulphonamide in cells and physiological fluids by isotope dilution liquid chromatography-tandem mass spectrometry.** *J Chromatogr B Analyt Technol Biomed Life Sci* 2009, **877:**3393-3399.
20. Monostori P, Wittmann G, Karg E, Túri S: **Determination of glutathione and glutathione disulfide in biological samples: an in-depth review.** *J Chromatogr B Analyt Technol Biomed Life Sci* 2009, **877:**3331-3346.
21. Steghens JP, Flourié F, Arab K, Collombel C: **Fast liquid chromatography-mass spectrometry glutathione measurement in whole blood: Micromolar GSSG is a sample preparation artifact.** *Journal of Chromatography B: Analytical Technologies in the Biomedical and Life Sciences* 2003, **798:**343-349.
22. Tomin T, Schittmayer M, Birner-Gruenberger R: **Addressing glutathione redox status in clinical samples by two-step alkylation with N-ethylmaleimide isotopologues.** *Metabolites* 2020, **10:**71.
23. Giustarini D, Colombo G, Garavaglia ML, Astori E, Portinaro NM, Reggiani F, Badalamenti S, Aloisi AM, Santucci A, Rossi R, et al: **Assessment of glutathione/glutathione disulphide ratio and S-glutathionylated proteins in human blood, solid tissues, and cultured cells.** *Free Radic Biol Med* 2017, **112:**360-375.
24. Samuelsson M, Vainikka L, Öllinger K: **Glutathione in the blood and cerebrospinal fluid: A study in healthy male volunteers.** *Neuropeptides* 2011, **45:**287-292.
25. Singh A, Kukreti R, Saso L, Kukreti S: **Oxidative Stress: A Key Modulator in Neurodegenerative Diseases.** *Molecules* 2019, **24:**1583.
26. Pollari E, Goldsteins G, Bart G, Koistinaho J, Giniatullin R: **The role of oxidative stress in degeneration of the neuromuscular junction in amyotrophic lateral sclerosis.** *Frontiers in Cellular Neuroscience* 2014, **8**.
27. GROUP TA: **ALSUntangled No. 52: Glutathione.** *Amyotrophic Lateral Sclerosis and Frontotemporal Degeneration* 2020, **21:**154-157.

28. Orrell RW, Lane RJM, Ross M: **A systematic review of antioxidant treatment for amyotrophic lateral sclerosis/motor neuron disease.** *Amyotrophic Lateral Sclerosis* 2008, **9:**195-211.
29. Vu LT, Bowser R: **Fluid-Based Biomarkers for Amyotrophic Lateral Sclerosis.** *Neurotherapeutics* 2017, **14:**119-134.
30. Verber NS, Shepheard SR, Sassani M, McDonough HE, Moore SA, Alix JJP, Wilkinson ID, Jenkins TM, Shaw PJ: **Biomarkers in Motor Neuron Disease: A State of the Art Review.** *Front Neurol* 2019, **10:**291-291.
31. Verber N, Shaw PJ: **Biomarkers in amyotrophic lateral sclerosis: a review of new developments.** *Curr Opin Neurol* 2020, **33:**662-668.
32. Thompson AG, Gray E, Verber N, Bobeva Y, Lombardi V, Shepheard SR, Yildiz O, Feneberg E, Farrimond L, Dharmadasa T, et al: **Multicentre appraisal of amyotrophic lateral sclerosis biofluid biomarkers shows primacy of blood neurofilament light chain.** *Brain Commun* 2022, **4:**fcac029.
33. Leckey CA, Coulton JB, Giovannucci TA, He Y, Aslanyan A, Laban R, Heslegrave A, Doykov I, Ammoscato F, Chataway J, et al: **CSF neurofilament light chain profiling and quantitation in neurological diseases.** *Brain Commun* 2024, **6:**fcae132.
34. Lee M, Hyun D, Jenner P, Halliwell B: **Effect of overexpression of wild-type and mutant Cu/Zn-superoxide dismutases on oxidative damage and antioxidant defences: relevance to Down's syndrome and familial amyotrophic lateral sclerosis.** *J Neurochem* 2001, **76:**957-965.
35. Chi L, Ke Y, Luo C, Gozal D, Liu R: **Depletion of reduced glutathione enhances motor neuron degeneration in vitro and in vivo.** *Neuroscience* 2007, **144:**991-1003.
